# Epidemiology of Ischemic Stroke and Hemorrhagic Stroke in Venoarterial Extracorporeal Membrane Oxygenation

**DOI:** 10.21203/rs.3.rs-3200908/v1

**Published:** 2023-07-31

**Authors:** Jaeho Hwang, Andrew Kalra, Benjamin L Shou, Glenn Whitman, Christopher Wilcox, Daniel Brodie, Akram M Zaaqoq, Roberto Lorusso, Ken Uchino, Sung-Min Cho

**Affiliations:** Johns Hopkins Hospital; Johns Hopkins Hospital; Johns Hopkins Hospital; Johns Hopkins Hospital; Mercy Hospital of Buffalo; Johns Hopkins University School of Medicine; University of Virginia; Maastricht University Medical Centre; Cleveland Clinic Lerner College of Medicine of Case Western Reserve University; Johns Hopkins Hospital

**Keywords:** Venoarterial ECMO, Stroke, Mortality, Trend

## Abstract

**Background:**

While venoarterial extracorporeal membrane oxygenation (VA-ECMO) provides lifesaving support for cardiopulmonary failure, complications may arise that increase mortality, with few studies focusing on ischemic/hemorrhagic stroke. We aimed to determine the trends of stroke incidence and mortality, associations with each other, and associations with total case volume at each Extracorporeal Life Support Organization (ELSO) center.

**Methods:**

Retrospective analysis of ELSO registry, including adult VA-ECMO patients from 534 international centers between 2012–2021, excluding extracorporeal cardiopulmonary resuscitation. Cochran-Armitage test and Poisson regression were used for trend analysis of stroke incidence and mortality. Kaplan-Meier curves, hazard functions, and multivariable logistic regression were used to study the impact of stroke on 90-day mortality.

**Results:**

Of 33,041 patients (median age = 58 years, female = 32%), 4% developed ischemic stroke, and 2% developed hemorrhagic stroke. Ischemic stroke incidence increased (×1.21/year, p < 0.0001), while hemorrhagic stroke incidence remained stable, and overall 90-day mortality declined (1.78%/year, p < 0.0001). Ischemic/hemorrhagic strokes were associated with increased overall 90-day mortality (OR = 3.29, 3.99 respectively, both p < 0.0001) after controlling for pre-selected covariates, including age, pre/post-cannulation lab values, ECMO duration, center volume, and on-ECMO complications. Total center volume was associated positively with ischemic/hemorrhagic stroke incidences (OR = 1.039, 1.053 per-additional-100-cases respectively, both p = 0.022), but inversely with 90-day mortality (OR = 0.909 per-additional-100-cases, p < 0.0001). Hazard of death was highest in the first several days of VA-ECMO.

**Conclusion:**

In VA-ECMO patients, while the reported ischemic stroke incidence steadily increased over time, 90-day mortality decreased. ELSO centers with higher case volumes reported greater stroke incidence, but lower mortality. Both ischemic/hemorrhagic strokes were associated with increased mortality.

## Background

Venoarterial (VA) extracorporeal membrane oxygenation (ECMO) is a form of temporary life support for patients with refractory cardio-circulatory or cardiopulmonary failure. It operates by extracting de-oxygenated blood from the patients’ venous system, and reinfusing oxygenated blood into the patients’ arterial system with adequate pressure. However, VA-ECMO support is not without risk, as an increasing number of studies throughout the years have reported on various neurological complications, which in turn are associated with higher mortality [1, 2]. Very few studies focusing specifically on the incidence of ischemic and/or hemorrhagic stroke as a complication of VA-ECMO, excluding extracorporeal cardiopulmonary resuscitation (ECPR) [3, 4]. Additionally, prior ECMO studies in the pediatric population revealed that ECMO centers with lower case volumes had higher mortality, though such studies in the adult ECMO patient population are more limited [5, 6]. Therefore, a robust, large-scale epidemiological study is needed to explore the incidence and outcomes related to ischemic and hemorrhagic stroke in adult patients supported with VA-ECMO.

We sought to characterize the trends regarding ischemic/hemorrhagic stroke across time and center volume, as well as the effects of stroke on mortality adjusting for known risk factors, with the aim to 1) determine the incidence and trend of ischemic strokes, hemorrhagic strokes, and mortality over the past 10 years; 2) determine the association between the total case volume and incidence of ischemic stroke, hemorrhagic stroke, or mortality at each Extracorporeal Life Support Organization (ELSO) center; and 3) determine the association between mortality and ischemic or hemorrhage stroke.

## Methods

### Study Design and Population

The ELSO registry is an international voluntary database that collects information on usage, indications, complications, and outcomes of ECMO support in adults and children from 534 centers worldwide currently. The registry collects data related to patient demographics, clinical characteristics, pre-ECMO conditions, hemodynamic and laboratory values before and during ECMO support, complications that occur during ECMO support, and outcomes such as survival at the time of hospital discharge. Diagnosis and medical history are reported according to the *International Classification of Diseases, 9th Revision* (ICD-9) and *10th Revision* (ICD-10) codes.

This study was a retrospective analysis of the ELSO registry database from 2012–2021. Inclusion criteria consisted of adult patients (≥ 18 years) who underwent VA-ECMO support for either cardiac or respiratory failure. Exclusion criteria consisted of pediatric patients (< 18 years), patients who underwent other types of ECMO, such as ECPR or venovenous (VV)-ECMO, and/or patients whose information related to ischemic/hemorrhagic stroke were missing. The period of 2012–2021 was chosen to assess the current, modern practices of ECMO support over the past decade. Pediatric patients were excluded, as their pathophysiology of conditions requiring ECMO often differs considerably from those in adults. ECPR and VV-ECMO were also excluded as their risk factors and mechanisms of acute brain injury differ from those of VA-ECMO. This study was approved by the local institutional review board.

### Data Collection and Definitions

For all included patients, the following data were collected from the ELSO registry: patient demographics, ECMO characteristics, pre- and post-ECMO cannulation laboratory values and hemodynamics, on-ECMO complications and their reported timings, including ischemic stroke, hemorrhagic stroke, and status at hospital discharge. For the variable of ischemic stroke, the ELSO registry terms “CNS diffuse ischemia”, defined as computed tomography (CT) or magnetic resonance imaging (MRI) demonstrating diffuse ischemic changes, and “CNS infarction”, defined as CT or ultrasound or MRI demonstrating localized ischemic change, were combined. For the variable of hemorrhagic stroke, three ELSO registry terms were combined: “CNS hemorrhage”, which was discontinued in 2018, as well as “intra/extra parenchymal CNS hemorrhage” and “intraventricular CNS hemorrhage”, which were newly introduced in 2018. Similarly, the timing of the detected strokes also began to be reported reliably in 2018 in the ELSO registry.

### Outcomes

Primary outcome was defined as 90-day mortality, while secondary outcome was defined as 30-day mortality.

### Statistical Analysis

Continuous variables were reported as medians with interquartile range (IQR). Categorical variables were reported as frequencies with percentages. For the patients’ ages, the ELSO registry records the data as a continuous variable with the exception of the age group “80 or older”. In this setting, the ages of patients “80 or older” were changed to 80, so that age could still be analyzed as a continuous variable. To assess the differences in the demographic information and ECMO-related variables between patients with and without strokes, t-test was used to compare the means of continuous variables, while χ^2^ test was used to compare proportions.

The Cochrane-Armitage test and Poisson regression were used to evaluate the trends over time (Aim 1). Scatterplots and associated locally estimated scatterplot smoothing (LOESS) curves were generated. Univariate logistic regression models were also created, with the total ELSO center volume as the independent variable, and the incidences of ischemic stroke, hemorrhagic stroke, 90-day mortality, and 30-day mortality as the dependent variables (Aim 2). Kaplan-Meier curves and hazard function curves were generated, and log-rank tests were performed (Aim 3). Cox-regression analysis was initially considered, however, ultimately unable to be performed, as the test for the proportional hazard assumption based on the Schoenfeld residuals revealed that this requirement was not met. Instead, a multivariable logistic regression model was generated with the dependent variable of 90-day (primary outcome) or 30-day mortality (secondary outcome).

Independent covariates chosen a priori consisted of patient demographics including age and sex; pre-ECMO blood gas values of arterial oxygen pressure (PaO_2_) and pH; post-cannulation 24-hour blood gas values of PaO_2_ (24h-PaO_2_) and pH (24h-pH); ECMO duration; and ECMO complications, including ischemic and/or hemorrhagic stroke, gastrointestinal hemorrhage, cardiac arrhythmia, pump failure, moderate-severe hemolysis (peak plasma hemoglobin > 50mg/dL or at least one occurrence of > 500mg/L during ECMO, sustained for ≥ 2 days), the requirement of neurosurgical intervention, renal replacement therapy, and/or cardiopulmonary bypass. These specific variables were chosen based on their medical relevance and the clinically hypothesized significance of their associations with mortality. Of note, the ELSO definitions of blood gas lab values were also updated in 2017 and 2018, such that pre-ECMO PaO_2_ and pH were changed from “worst” values to “closest to” ECMO start time, and 24h-PaO_2_ and 24h-pH were changed from “best” values to “closest to 24 hours” of ECMO. Accordingly, the logistic regression model assessing mortality was generated only with patient data reported from 2018 or later. None of these variables had missing data that exceeded 30%. p-values < 0.05 were considered statistically significant. All statistical analyses were performed using R Studio (R 4.1.2, www.r-project.org).

## Results

Overall, 34,734 VA-ECMO patients were queried from the 2012–2021 ELSO database. Of those, 1,693 patients were later converted to different modes of ECMO, and thus excluded from analysis, resulting in 33,041 patients. 20,297 had complete mortality data, of which 12,327 were from 2018–2021 (**Additional File 1**). Table 1 summarizes the variables related to patient demographics, ECMO, and mortality. Among the patients with complete mortality data, 96% (n=19,389) had no stroke, 3% (n=659) developed ischemic strokes, 1% (n=287) developed hemorrhagic strokes, and 0.2% (n=38) developed both ischemic and hemorrhagic strokes during ECMO support. The median detection timing was 3.04 days for ischemic strokes and 3.79 days for hemorrhagic strokes since ECMO cannulation. The 90-day mortality was higher for patients with ischemic strokes than those without (65% vs 36% respectively, p<0.0001, c^2^ test). Similarly, the 90-day mortality was higher for patients with hemorrhagic strokes than those without (72% vs 37% respectively, p<0.0001, c^2^ test). Many ECMO-related variables were found to be significantly different between patients with ischemic or hemorrhagic stroke and those without (Table 1). Notably, patients who developed ischemic stroke experienced more on-ECMO complications, compared to those without, including longer duration of ECMO support (6.17 vs 4.75 days respectively, p<0.0001, t-test), more neurosurgical interventions (1% vs 0.07% respectively, p<0.0001, c^2^ test), and more cardiac arrhythmias (20% vs 11% respectively, p<0.0001, c^2^ test). This was similar to patients who developed hemorrhagic strokes, compared to those without, including longer duration of ECMO support (6.33 vs 4.79 days respectively, p<0.0001, t-test), more neurosurgical interventions (4% vs 0.04% respectively, p<0.0001, c^2^ test), and more gastrointestinal hemorrhage (9% vs 3% respectively, p<0.0001, c^2^ test).

### Trend of VA-ECMO associated ischemic stroke, hemorrhagic stroke, and mortality

**Figure 1:**
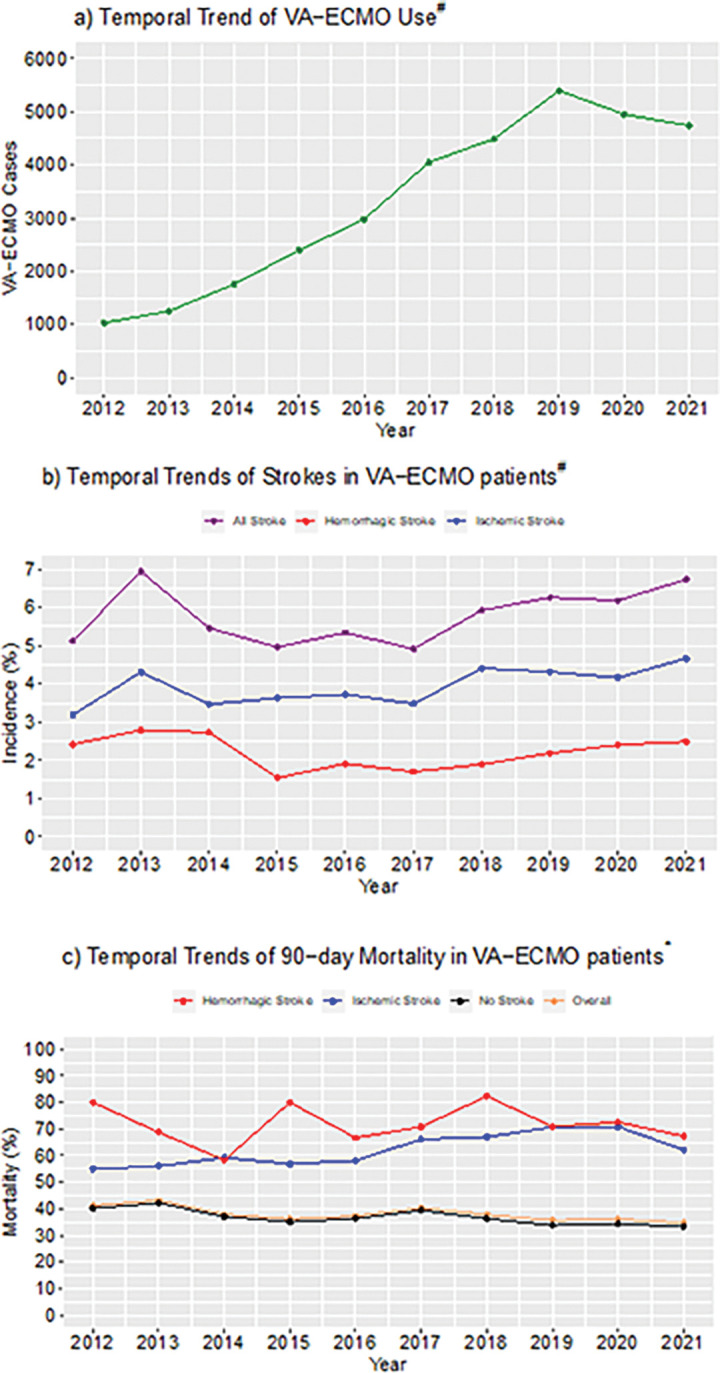
Temporal Trends of VA-ECMO, Strokes, and 90-day Mortality Abbreviations: ^#^: All 33,041 cases; *: 20,297 cases with complete mortality data; VA-ECMO: venoarterial extracorporeal membrane oxygenation

Fig. 1a shows the trend of VA-ECMO cases submitted to the ELSO registry database. The number of cases has generally increased during the past decade, from 1,034 cases in 2012 to 4,737 cases in 2021, corresponding to a roughly 1.18 times increase per year (p<0.0001, Poisson regression). Fig. 1b depicts the temporal trends of the reported incidence of ischemic and hemorrhagic strokes. The incidence of hemorrhagic stroke did not change overall between 2012 and 2021 (p=0.41, Cochran-Armitage). However, the incidence of ischemic stroke increased (p=0.002, Cochran-Armitage), with the number of cases increasing roughly 1.21 times per year (p<0.0001, Poisson regression). Fig. 1c and **Additional File 2** illustrate the temporal trends of 90-day and 30-day mortality, respectively. The overall 90-day mortality declined (p<0.0001, Cochran-Armitage) by 1.78% per year between 2012 and 2021 (p=0.0003, Poisson regression). Similarly, 90-day mortality of patients without strokes also declined (p<0.0001, Cochran-Armitage) by 2.02% per year (p<0.0001, Poisson regression). In contrast, 90-day mortality of patients with hemorrhagic stroke did not significantly change over time (p=0.85). While the 90-day mortality of patients with ischemic stroke showed a potential trend towards increased mortality over time, this did not reach statistical significance (p=0.053).

### Association of ELSO center volume and incidence of ischemic stroke, hemorrhagic stroke, or mortality

**Figure 2:**
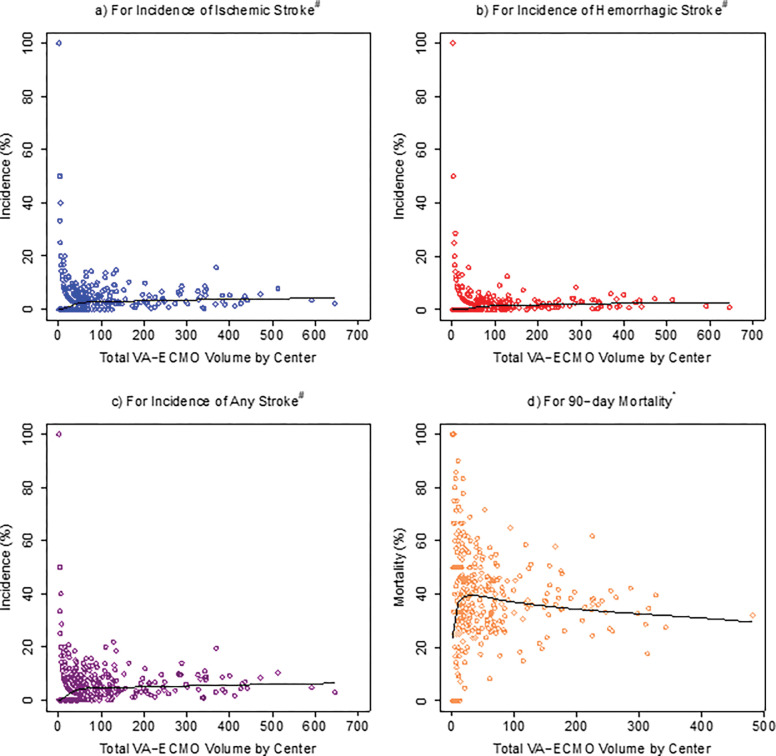
Associations Between ELSO Center Volume and Incidence of Strokes or 90-day Mortality Abbreviations: ^#^: All 33,041 cases; *: 20,297 cases with complete mortality data, VA-ECMO: venoarterial extracorporeal membrane oxygenation

Fig. 2 and **Additional File 3** demonstrate the associations between the total case volume of each ELSO center and its incidence of strokes or mortality. The total case volumes of each center ranged from 1 to 646. Univariable logistic regression revealed that for each additional 100 cases of VA-ECMO that an ELSO center has experienced, the incidences of strokes increased by 3.93% for ischemic stroke (p=0.022), 5.83% for hemorrhagic stroke (p=0.022), and 3.67% for any strokes (p=0.011). In contrast, the 90-day mortality decreased by 9.07% (p<0.0001) per each additional 100 cases of VA-ECMO at an ELSO center. This statistical significance for total center volume became even greater in the multivariable logistic regression models for 90-day and 30-day mortality (Table 2, **Additional File 4**).

### Ischemic and hemorrhage stroke as risk factors for mortality

**Figure 3:**
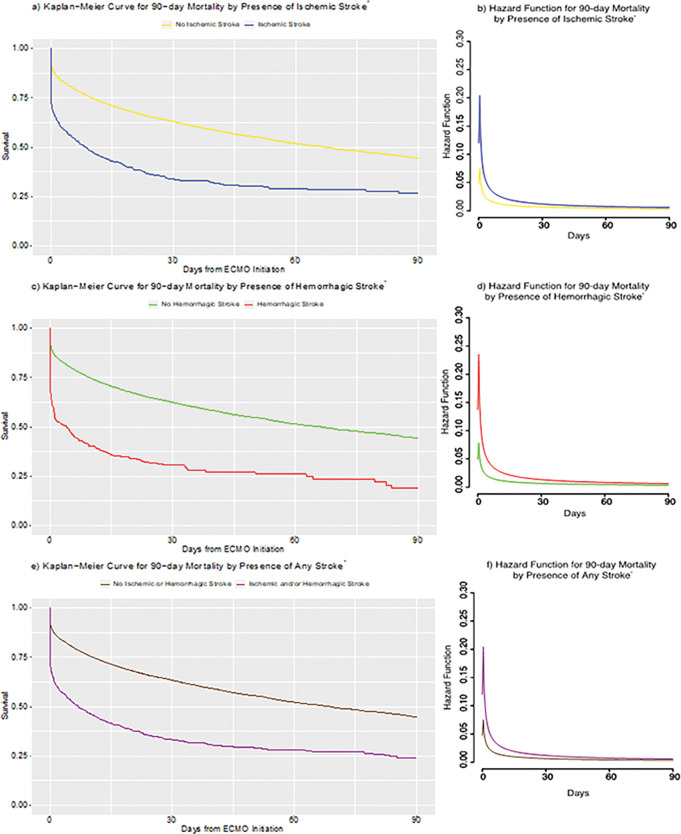
Kaplan-Meier Curves and Hazard Functions for 90-day Survival by Presence or Absence of Strokes Abbreviations: *: 20,297 cases with complete mortality data; ECMO: extracorporeal membrane oxygenation

Fig. 3 and **Additional File 5** show the overall survival curves and hazard functions of VA-ECMO patients with or without strokes. Median 90-day survival times were significantly different between patients who developed strokes and those who did not: 8.54 vs 67.17 days in the presence or absence of ischemic stroke respectively (p<0.0001, log-rank test); 4.00 vs 66.20 days in the presence or absence of hemorrhagic stroke respectively (p<0.0001, log-rank test); and 7.00 vs 68.80 days in the presence or absence of any stroke respectively (p<0.0001, log-rank test).

The multivariable logistic regression models for 90-day and 30-day mortality revealed numerous, significantly associated variables (Table 2, **Additional File 4**). In particular, the odds of 90-day mortality were higher for patients with ischemic stroke than those without (odds ratio=3.29, p<0.0001, logistic regression). This was also similar to the case of 90-day mortality of patients with hemorrhagic stroke compared to those without (odds ratio=3.99, p<0.0001, logistic regression).

## Discussion

This study is the largest to date that assesses the associations of mortality and strokes in the context of other ECMO-related variables, as well as the trends of stroke incidence over 10 years in VA-ECMO. The ELSO registry database has grown markedly, with the number of reported cases increasing nearly 5-fold over the past decade, continuing the trend identified in the ELSO study of VA-ECMO from 1992–2013 [2]. Encouragingly, the overall 90-day and 30-day mortality rate of VA-ECMO patients have steadily down-trended over time (Fig. 1, **Additional File 2**). This is also consistent with the survival trends of ECMO patients in the ELSO database since its inception in 1989, which has steadily risen over time from <1% survival rate [7]. However, the survival for patients who developed ischemic and/or hemorrhagic strokes has not improved. While the incidence of ischemic stroke appears to have mildly increased over time, this may be in the setting of more recognition and vigilance regarding ECMO-related complications, leading to more detection of ischemic strokes, rather than worsening ECMO management. For example, a study conducted at Johns Hopkins Hospital demonstrated that a standardized neuromonitoring protocol for ECMO patients led to higher detection of acute brain injury, yet improved patient outcomes [1]. Alternatively, the increasing incidence of strokes as a complication of VA-ECMO over time may be due to VA-ECMO being offered to a progressively wider patient population, including those who may be more critically ill than in prior years, as ECMO use and practice have evolved over time with more experience and standardized ECMO care.

Additionally, the more experienced ELSO centers generally had lower mortality, but mildly increased detections of ischemic and/or hemorrhagic strokes (Fig. 2, **Additional File 3**). In contrast, the ELSO centers with very low total case volumes had incidences of stroke and mortality that ranged broadly (0–100% for both), which is likely explained by high variance in the setting of nearly half of the registered ELSO centers having total center volume fewer than 10 cases, and over 100 centers with two or fewer cases. Notably, the total case volume of ELSO centers was also inversely related to 90-day and 30-day mortality in the multivariable logistic regression models (Table 2, **Additional File 4**). One possible explanation is that more experienced ELSO centers have more established infrastructure and protocols for ECMO patients, thereby being prepared and able to detect more complications, which may then lead to improved survival with timely interventions.

In general, however, the presence of ischemic and/or hemorrhagic stroke was significantly associated with increased 90-day and 30-day mortality (Fig. 3, **Additional File 5**). The hazard of death was highest in the first week of ECMO support, by which time 82% of all ischemic strokes and 78% of all hemorrhagic strokes had occurred, then plateaued in the subsequent weeks. Hazards were even higher for patients who developed strokes early on as a complication of ECMO compared to those who did not. Prior literature evaluating the associations between mortality and strokes are limited, both in quantity and the mostly single-center nature of the studies. Overall, these studies have shown mixed results, with some reporting hemorrhagic stroke, but not ischemic stroke, as a risk factor for increased mortality during ECMO support [8,9], while others also reporting ischemic stroke to be a risk factor [10–13]. In this context, our multi-center study contributes further evidence that both ischemic and hemorrhagic strokes are associated with increased 90-day and 30-day mortality.

Risk factors other than ischemic or hemorrhagic strokes that contributed to higher mortality in the logistic regression included older age, lower total center volume, longer ECMO duration, and presence of other on-ECMO complications, namely gastrointestinal hemorrhage, moderate-severe hemolysis, and renal replacement therapy (Table 2, **Additional File 4**). The positive association between age and mortality is consistent with prior studies [14]. Additionally, patients who require longer ECMO support are likely more prone to developing on-ECMO complications, which lead to poorer prognosis, compared to those without on-ECMO complications. Several of the significant covariates in our model for mortality have also been previously shown to be risk factors for ischemic and/or hemorrhagic strokes in our prior studies using the ELSO database, including ECMO duration, 24-hour PaO_2_, renal replacement therapy, and moderate-severe hemolysis [3,4].

### Limitations

This study has several limitations. First, the ELSO data has missing information, though analyses were restricted to variables with missing data of less than 30%. However, the major advantage of the ELSO registry data is its large sample size that allows for higher statistical power, hundreds of available variables, and the multi-institutional nature that allows for generalizability of the study. Second, the ECMO protocols of the numerous ELSO centers are not necessarily standardized, which may lead to some differences in diagnoses of complications, such as how and when strokes were diagnosed and/or management decisions, such as when neurosurgical interventions were performed. Nevertheless, given that these medical decisions were made based on best clinical judgment and necessity, these differences were considered to be relatively minor overall. Third, information regarding the size and severity of the strokes during ECMO support were unavailable in the database, and thus could not be accounted for in the analysis.

## Conclusions

Over the past decade, while the reported incidences of ischemic and hemorrhagic stroke in VA-ECMO patients have progressively increased, 90-day mortality has decreased, possibly explained by the increased awareness, detection, and management of strokes as a potential complication of VA-ECMO. ELSO centers with higher total case volumes also tended to report higher rates of stroke detection, but lower mortality. The development of ischemic and hemorrhagic strokes during VA-ECMO were both, independent risk factors for higher mortality after adjusting for age, sex, total center volume, pre-ECMO and post-cannulation 24-hour blood gas values, ECMO duration, and other on-ECMO complications, including gastrointestinal hemorrhage, cardiac arrhythmia, pump failure, moderate-severe hemolysis, and requirement of neurosurgical intervention, renal replacement therapy, and/or cardiopulmonary bypass. Continued improvements in the standardized practices of preventing, detecting, and treating the on-ECMO complications of ischemic and hemorrhagic strokes as will further benefit the VA-ECMO patient population.

## Figures and Tables

**Table 1:** Patient demographics and ECMO variables for ECMO-associated ischemic stroke and hemorrhagic stroke

Variable	Ischemic Stroke (n=659)	No Ischemic Stroke (n=19,638)	p	Hemorrhagic Stroke (n=287)	No Hemorrhagic Stroke (n=20,010)	p
Age (years)	58.1 (46.4–65.4)	57.5 (45.3–66.2)	0.61	57.9 (46.1–65.6)	57.5 (45.3–66.2)	0.82
Female	234 (36%)^f^	6,310 (32%)^g^	0.09	114 (40%)^h^	6,430 (32%)^i^	**0.01**
Total Center Volume	123 (56–234)	123 (55–225)	0.31	127 (61–237)	123 (55–225)	0.25
Year of ECMO						
2012	20 (3%)	581 (3%)	0.35	10 (3%)	591 (3%)	0.17
2013	25 (4%)	718 (4%)		16 (6%)	727 (4%)	
2014	27 (4%)	1,001 (5%)		12 (4%)	1,016 (5%)	
2015	51 (8%)	1,352 (7%)		15 (5%)	1,388 (7%)	
2016	55 (8%)	1,716 (9%)		21 (7%)	1,750 (9%)	
2017	59 (9%)	2,365 (12%)		24 (8%)	2,400 (12%)	
2018	100 (15%)	2,726 (14%)		34 (12%)	2,792 (14%)	
2019	123 (19%)	3,249 (17%)		55 (19%)	3,317 (17%)	
2020	96 (15%)	2,975 (15%)		51 (18%)	3,020 (15%)	
2021	103 (16%)	2,955 (15%)		49 (17%)	3,009 (15%)	
Pre-ECMO ABG						
pH (2012–2016)^a^	7.25 (7.12–7.35)^f^	7.30 (7.19–7.39)^g^	**0.002**	7.25 (7.13–7.32)^h^	7.30 (7.19–7.39)^i^	**0.004**
pH (2017–2021)^b^	7.28 (7.17–7.37)^f^	7.30 (7.21–7.39)^g^	**<0.001**	7.29 (7.20–7.36)^h^	7.30 (7.21–7.39)^i^	**0.04**
PaO_2_ (2012–2016)^a^	95 (59–216)^f^	96 (64–197)^g^	0.35	97 (68–159)^h^	96 (64–197)^i^	0.32
PaO_2_ (2017–2021)^b^	100 (68–254)^f^	116 (74–232)^g^	0.63	107 (76–218)^h^	116 (74–233)^i^	0.25
ABG at 24 hours						
pH (2012–2017)^c^	7.42 (7.38–7.45)^f^	7.42 (7.38–7.47)^g^	0.50	7.41 (7.36–7.45)^h^	7.42 (7.38–7.47)^i^	0.18
pH (2018–2021)^d^	7.43 (7.37–7.47)^f^	7.43 (7.38–7.47)^g^	0.29	7.43 (7.39–7.48)^h^	7.43 (7.38–7.47)^i^	0.09
PaO_2_ (2012–2017)^c^	149 (92–254)^f^	143 (94–238)^g^	0.62	157 (91–299)^h^	143 (94–238)^i^	0.15
PaO_2_ (2018–2021)^d^	132 (88–270)^f^	134 (91–222)^g^	0.08	129 (86–207))^h^	134 (91–224)^i^	0.81
Days on ECMO Support	6.17 (3.71–10.04)	4.75 (2.71–7.67)	**<0.001**	6.33 (3.71–10.81)	4.79 (2.71–7.71)	**<0.001**
Timing of Stroke (days) (2018–2021)	3.04 (1.29–6.29)^f^	N/A	N/A	3.79 (1.25–6.88)	N/A	N/A
Mortality						
90-day Mortality	427 (65%)	7,123 (36%)	**<0.001**	206 (72%)	7,344 (37%)	**<0.001**
30-day Mortality	411 (62%)	6,339 (32%)	**<0.001**	195 (68%)	6,555 (33%)	**<0.001**
ECMO Complications						
Cardiac Arrhythmia	129 (20%)	2,112 (11%)	**<0.001**	52 (18%)	2,189 (11 %)	**<0.001**
CPB	166 (25%)	4,172 (21%)	**0.02**	54 (19%)	4,284 (21%)	**0.32**
GI Hemorrhage	48 (7%)	601 (3%)	**<0.001**	27 (9%)	622 (3%)	**<0.001**
Hemolysis (Mod-Sev)	22 (3%)	261 (1%)	**<0.001**	7 (2%)	276 (1 %)	0.21
Neurosurgical Int (2018–2021)^e^	5 (1 %)	8 (0.07%)	**<0.001**	8 (4%)	5 (0.04%)	**<0.001**
Pump	7 (1 %)	100 (0.5%)	0.10	4 (1 %)	103 (0.5%)	0.10
Failure						
RRT	237 (36%)	4,509 (23%)	**<0.001**	94 (33%)	4,652 (23%)	**<0.001**

All data are presented as n (%) for categorical variables, and median (interquartile range) for continuous variables.

Abbreviations:

a: “worst” values;

b: “closest” value to ECMO start time;

c: “best” values;

d: “closest” value to 24-hours of ECMO;

e: Used sample size of patients in years 2018-2021 as denominator;

f: Missing values for variables in the “Ischemic Stroke” group: Sex (n=3), pre-ECMO pH (n=175), pre-ECMO PaO2 (n=188), 24-hour-pH (n=43), 24-hour-PaO2 (n=53), timing of stroke (n=9);

g: Missing values for variables in the “No Ischemic Stroke” group: Sex (n=176), pre-EMCO pH (n=4,995), pre-EMCO PaO2 (n=5,584), 24-hour-pH (n=2,644), 24-hour-PaO_2_ (n=3,247);

h: Missing values for variables in the “Hemorrhagic Stroke” group: pH (n=65), PaO_2_ (n=70), 24h-pH (n=30), 24h-PaO_2_ (n=34);

i: Missing values for variables in the “No Hemorrhagic Stroke” group: Sex (n=179), pH (n=5,105), PaO_2_ (n=5,702), 24h-pH (n=2,657), 24h-PaO_2_ (n=3,266); ABG: arterial blood gas; CPB: cardiopulmonary bypass; ECMO: extracorporeal membrane oxygenation; GI: gastrointestinal; Int: intervention; Mod: moderate; N/A: not applicable; RRT: renal replacement therapy; Sev: severe

**Table 2:** Logistic Regression of Variables Associated with 90-day Mortality^

	Odds ratio	95% CI	p-value
**Age**	**1.04**	[1.03–1.04]	**<1×10^−4^**
Female	1.07	[0.96–1.19]	0.20
**Total Center Volume**	**0.998**	[0.997–0.999]	**<1×10^−4^**
Pre-ECMO ABG			
pH	2.19×10^−10^	[9.79×10^−24^−3.01×10^3^]	0.15
PaO_2_	1.00	[0.9991–1.000]	0.36
ABG at 24 hours of ECMO support			
pH	1.81×10^−10^	[1.51×10^−23^−1.34×10^3^]	0.14
**PaO_2_**	**1.001**	[1.001–1.002]	**<1×10^−4^**
Pre-ECMO pH × 24h-pH^a^	17.69	[0.30–1.12×10^3^]	0.17
Pre-ECMO PaO_2_ × 24h-PaO_2_^b^	1.00	[1.00–1.00]	0.54
**ECMO Duration (days)**	**1.02**	[1.01–1.03]	**<1×10^−4^**
ECMO Complications			
Cardiac Arrhythmia	1.08	[0.93–1.25]	0.30
Cardiopulmonary Bypass	1.04		0.50
**Gastrointestinal Hemorrhage**	**1.71**	[1.32–2.22]	**<1×10^−4^**
**Hemolysis (Moderate-Severe)**	**1.37**	[1.01–1.84]	**0.04**
**Hemorrhagic Stroke**	**3.99**	[2.60–6.28]	**<1×10^−4^**
**Ischemic Stroke**	**3.29**	[2.52–4.33]	**<1×10^−4^**
Neurosurgical Intervention	1.94	[0.51–8.14]	0.33
Pump Failure	1.15	[0.57–2.30]	0.69
**Renal Replacement Therapy**	**2.02**	[1.81–2.26]	**<1×10^−4^**
**Ischemic Stroke × Hemorrhagic Stroke^c^**	**0.17**	[0.06–0.47]	**5.76×10^−4^**

Abbreviations:

^ :12,327 cases with complete mortality data from 2018–2021;

a : interaction term between pre-ECMO pH and 24 hour pH;

b : interaction term between pre-ECMO PaO_2_ and 24 hour PaO_2_;

c : interaction term between ischemic stroke and hemorrhagic stroke; ABG: arterial blood gas; CI: confidence interval; ECMO: extracorporeal membrane oxygenation

## Data Availability

The data that support the findings of this study are available from the Extracorporeal Life Support Organization (ELSO), but restrictions apply to the availability of these data, which were used under license for the current study, and so are not publicly available. Data are however available from the authors upon reasonable request and with permission of ELSO.

## Abbreviations

24h-PaO_2_: post-cannulation 24-hour arterial oxygen pressure
24h-pH: post-cannulation 24-hour blood gas pH
CT: computed tomography
ECMO: extracorporeal membrane oxygenation
ECPR: extracorporeal cardiopulmonary resuscitation
ELSO: Extracorporeal Life Support Organization
ICD-9: International Classification of Diseases, 9th Revision
ICD-10: International Classification of Diseases, 10th Revision
IQR: interquartile range
LOESS: locally estimated scatterplot smoothing
MRI: magnetic resonance imaging
PaO_2_: arterial oxygen pressure
VA: venoarterial
VV: venovenous
